# Pain Management in Pancreatic Cancer

**DOI:** 10.3390/cancers3010043

**Published:** 2010-12-24

**Authors:** Mariam Hameed, Haroon Hameed, Michael Erdek

**Affiliations:** 1 Department of Physical Medicine and Rehabilitation, School of Medicine, Johns Hopkins University, 600 N. Wolfe St., Phipps 160, Baltimore, MD 21287, USA; E-Mails: mhameed1@jhmi.edu (M.H.); hhameed1@jhmi.edu (H.H.); 2 Division of Pain Medicine, Department of Anesthesia and Critical Care Medicine, Johns Hopkins University, School of Medicine, 550 North Broadway St., Suite 301, Baltimore, MD 21205, USA

**Keywords:** pancreatic cancer, neurolytic celiac plexus block, intrathecal therapy, opioids, pain

## Abstract

A majority of pancreatic cancer patients present with pain at the time of diagnosis. Pain management can be challenging in light of the aggressive nature of this cancer. Apart from conventional pharmacotherapy, timely treatment with neurolytic celiac plexus block (NCPB) has been shown to be of benefit. NCPB has demonstrated efficacious pain control in high quality studies with analgesic effects lasting one to two months. NCPB has also shown to decrease the requirements of narcotics, and thus decrease opioid related side effects. Another option for the control of moderate to severe pain is intrathecal therapy (IT). Delivery of analgesic medications intrathecally allows for lower dosages of medications and thus reduced toxicity. Both of the above mentioned interventional procedures have been shown to have low complication rates, and be safe and effective. Ultimately, comprehensive pancreatic cancer pain management necessitates understanding of pain mechanisms and delivery of sequential validated therapeutic interventions within a multidisciplinary patient care model.

## 1. Introduction

The incidence of pancreatic cancer has remained fairly constant throughout the past three decades, affecting approximately 30,000 people each year. The five year survival rate continues to be dismal, with approximately less than 5% surviving, making it the fourth leading cause of cancer-related deaths [1]. This in part is attributed to advanced disease at presentation, making surgical cure unlikely. Median survival of patients with unresectable pancreatic adenocarcinoma is 5.8 months [2], while median survival in resectable disease is 12 to 15.9 months [3]. The cornerstone of management of these patients is palliative care. Nearly 75% of patients suffer from pain at the time of diagnosis, with over 90% of patients in advanced stages [4]. Historically, management with non-steroidal anti-inflammatory agents and narcotics were the mainstay of therapy. In recent years, celiac plexus blocks and neurolysis, splanchnicectomy, and intrathecal therapies have been used to combat severe pain, at times resulting in decreased requirement of drugs and their unwanted side effects.

## 2. Pancreatic Cancer Pain

Pain in pancreatic cancer may be visceral, somatic, or neuropathic in origin. Pain is produced by tissue damage, inflammation, ductal obstruction, and infiltration. Visceral nociceptive signals caused by damage to the upper abdominal viscera are carried along sympathetic fibers which travel to the celiac plexus nerves and ganglia which are found at the T12-L2 vertebral levels [5,6] anterolateral to the aorta near the celiac trunk. From here the signals are transmitted through the splanchnic nerves to T5-T12 dorsal root ganglia, and on to the higher centers of the central nervous system. Somatic and neuropathic pain may arise from tumor extension into the surrounding peritoneum, retroperitoneum, bones and in the latter case, nerves such as the lumbosacral plexus. It should also be noted that other types of pain may arise in consequence to therapeutic interventions such as post-chemoradiation syndromes causing mucositis and enteritis [7].

## 3. Pharmacotherapy

Individualized pharmacotherapy is considered the mainstay of treatment for pain control in cancer patients. A standardized approach for analgesic drug regimens administered for the control of chronic cancer pain has been put forth by the World Health Organization (WHO) in the form of an “analgesic ladder” [8]. This stepwise approach is based on the severity of pain and less on the pathophysiologic process of pain. It is essential that physicians keep in mind the multiple types of pain generating processes (visceral, somatic, and neuropathic) in pancreatic cancer in order to increase the efficacy of available therapeutic modalities. The analgesic ladder mainly uses non-steroidal anti-inflammatory drugs (NSAID) and opioids. Opioids exhibit additive analgesia and should be administered with care. Further, it is important to note that the plasma half-life of some opioids do not correlate with duration of analgesia. For example, methadone has a half-life of 8-59 hours but analgesic duration lasts for 4–8 hours. Tables 1 and 2 detail commonly prescribed analgesics. As pancreatic cancer patients often suffer from gastrointestinal dysfunction, medications may alternatively be given by the rectal, transdermal, subcutaneous, intravenous or intrathecal routes. It has been suggested that nearly one-third of cancer patients will require up to four routes of opioid administration [9]. Additionally, adjuvant medications, which help control the medication side effects such as constipation and nausea, those which enhance analgesic effects like steroids, or those which control fear and anxiety such as antidepressants, can be added at any step on the WHO ladder [7].

A more comprehensive oncologic pain management guideline was put forth by the National Comprehensive Cancer Network (NCCN) [10] and is discussed in greater detail below. They systematically target therapy to those patients who are opioid naïve, opioid tolerant, and who require ongoing care. They also take into account procedure-related pain and anxiety, neuropathic pain, treatment of side effects from narcotics, and the use of non-pharmacologic therapies. Patients at all pain levels are recommended to receive psychological support, education, non-pharmacologic interventions, e.g., heat/ice, massage, cognitive behavioral therapy, or relaxation training; and treatment of side effects from pharmacotherapy.

### 3.1. Opioid Naïve Patients

For control of mild pain, non-steroidal anti-inflammatory drugs (NSAID) are the most appropriate first-line agents. A sufficient trial of each medication should be undertaken, which would include giving the drug at significant dosages at regular intervals before switching to another drug [11]. An in-depth review published by the Agency for Healthcare Research and Quality (AHRQ) cites evidence showing little difference in the analgesic efficacy between NSAIDS and weak opioids [12]. However, NSAID use may be limited due to inability to obtain additive analgesia past certain dosages and to the development of side effects like gastrointestinal bleeding or nephrotoxicity [11]. For patients suffering from moderate to severe pain or who are unable to tolerate non-opioid analgesics, the addition of an opioid, such as hydrocodone, is indicated. When starting opioids (e.g., morphine sulfate) in naïve patients with moderate to severe pain, pain should be re-evaluated at frequent intervals and titrated for two-to-three cycles as needed until adequate pain control is achieved [10].

### 3.2. Opioid Tolerant Patients [10]

Opioid tolerant patients often require larger dosages of opioids. When pain levels reach ≥4 on the numeric rating scale, the NCCN recommends giving 10-20% of the total dose required in the previous 24 hours to control moderate to severe pain. Dosages may then be titrated upwards by 50–100% if the pain scores continue to be unchanged or increased. Similar to the above, assessments of pain relief are checked at frequent intervals and titrated for two-to-three cycles. Ongoing care in opioid tolerant patients includes continued titration for moderate pain, and re-evaluation of the working diagnosis and current medications (analgesics and adjuvants) prescribed, as well as consideration of involvement of pain specialists for those with severe pain.

### 3.3. Ongoing Patient Care [10]

At each routine follow-up visit, an assessment of the patient's goals of function and comfort, adherence to the pain plan and requirement of analgesics will help direct further management. It is important to ensure that the patient has sufficient access to medications and barriers to therapy are overcome. Should the patient continue to have severe pain, referral to a pain specialist may be necessary to incorporate interventional strategies, such as ganglion blocks or intrathecal therapies [13] to mitigate pain. Additionally, proper communication between involved health care providers within a multidisciplinary team underlies efficacious management.

### 3.4. Adjuvant Medications

In an effort to enhance analgesia and mitigate opioid side effects, adjuvant medications may be used. Optimization of analgesia in certain conditions may require medications such as corticosteroids for nerve compression or inflammatory pain, anticonvulsants and gabapentin for neuropathic pain, or bisphosphonates or chemotherapy for bone pain. Additional antineoplastic therapy such as hormones or radiation may also be beneficial in localized painful lesions. With regard to opioid side effects, stool softeners and laxatives for constipation, diphenhydramine for pruritus, and serotonin-antagonists like ondansetron for nausea are often used.

## 4. Celiac Plexus Block and Neurolysis

### 4.1. History

Since its first description in 1914 by Max Kappis, celiac plexus block (CPB) and neurolysis (CPN) have been therapeutic tools for the management of pancreatic pain [14]. Though the terms celiac plexus block and neurolysis are often used synonymously; CPB is the transient interruption of the celiac plexus when injected with a local anesthetic, whereas CPN is the prolonged interruption of the plexus via chemical ablation with alcohol or phenol (often given with a local anesthetic). Despite numerous positive retrospective studies, case series reports and supportive reviews [5,15]; the efficacy of neurolytic celiac block was called into question [16]. This was due to the recognition of major methodological deficiencies in existing prospective studies, including limited data collection leading to difficulty in deriving solid conclusions from individual, as well as when comparing studies; and ultimately, a lack of well-designed prospective randomized controlled trials (RCT). However, in the last 20 years, a number of RCTs and case series studies [15] have shown the benefit of neurolytic celiac plexus block (NCPB) in controlling chronic pancreatic cancer pain, as evidenced by both reduced pain scores and decreased post-procedural opioid consumption. Table 3 elaborates on the RCTs and their methodological quality using the AHRQ scoring system.

### 4.2. Techniques

Various techniques are used to achieve CPN, and can be performed blindly or under radiologic guidance by the use of fluoroscopy, CT-guidance, or endoscopic ultrasound. Approaches include the classic posterolateral or retrocrural approach, the transcrurual approach, the anterior approach, and intraoperative splanchnicectomy [17]. Ischia [18] and colleagues performed a prospective randomized trial comparing the transaortic, retrocrural, and bilateral chemical splanchnicectomy (Boas' approach) procedures. Their results showed that there was no difference in analgesic efficacy with respect to recurrent and residual (celiac or nonceliac) pain between the three, and that neurolytic celiac plexus block gave complete visceral pain relief in 70–80% of patients immediately and in up to 60–75% of patients until death. In contrast, when Suleyman *et al.* [19] compared the transaortic celiac plexus block with splanchnic nerve block in a prospective randomized trial in patients with carcinoma of the body and tail of the pancreas, they found that splanchnicectomy provided greater pain relief and decreased opioid consumption when compared to NCPB. Although there have been only a few published case series and a retrospective study [20-23] regarding use of endoscopic-ultrasonography (EUS) guided CPN since the first report in 1995 [19], a recent review evaluated EUS-guided CPN to be 72.54% effective in pancreatic cancer patients [24]. Further, EUS-guided CPB and CPN have also been reported to have low morbidity, with complication rates at 1.6% and 3.2%, respectively [25].

### 4.3. Efficacy of Neurolytic Celiac Plexus Block

Numerous studies have successfully demonstrated the efficacy of NCPB to control pancreatic cancer pain. Lillemoe *et al.* [26] conducted the first randomized controlled double-blinded trial studying the benefits of intraoperative splanchnicectomy with alcohol *versus* saline placebo at time of exploratory laparotomy for biopsy, staging, and/or palliative gastrointestinal bypass. All patients received postoperative pharmacotherapy at the discretion of their respective treating physicians. Patients were followed up at two month intervals until death. In this study, patients with no pre-existing pain were noted to have increasing pain scores until death; however, patients who underwent splanchnicectomy had a longer duration of pain free interval (7.2 months, compared to 3.0 months of placebo, p < 0.0001), and fewer required significant opioid doses (set at >10 mg IM morphine) compared to placebo (46% compared to 68% of placebo, p < 0.05). Similar results were found in those with pre-existing pain. Overall, they suggested that three-to-four months of mild pain might occur before return to severe pain. Although some patients in both groups required rescue block by percutaneous NCPB; time to rescue was significantly longer in the intervention group.

A more recent double-blinded RCT by Wong *et al.* demonstrated a greater reduction in pain scores for those patients post-NCPB in the first week after randomization [27]. In the first six weeks, significantly fewer patients reported moderate to severe pain (rated as ≥5/10) in the neurolysis group *versus* those in the pharmacotherapy only group (14% *vs.* 40%, p = 0.005). Although fewer patients in the neurolysis group required rescue blocks (6% *vs.* 20% in the pharmacotherapy only group), this finding was not significant (p = 0.07). Similarly, a significant reduction of pain scores have been reproduced in other RCTs: one reported efficacy for up to four weeks [28], and another [29] showed NCPB was effective in abolishing visceral celiac-pain in all treated patients in the immediate assessment period (24–48 hours), and in 10 of 12 patients until death (only one required no further medication).

A meta-analysis [15] of 24 studies of CPB in abdominal cancers assessing short- and long-term outcomes found 87% of 304 patients (in studies restricted to pancreatic cancer) benefited in the short-term period. Most studies were able to provide long term outcomes (≥3 months) from fewer patients. Out of 273 patients with long term follow-up available, nearly 90% had partial or complete pain relief. At the time of death, merged data in six studies with 53 mixed abdominal cancer patients showed 73% had partial to complete pain relief within three months, and 92% beyond three months.

### 4.4. Opioid Consumption

Late in 1993, after Lillemoe published his results, an Italian group [30] published a randomized unblind study of 20 patients, split into two groups of 10 each, who were followed until death and pain scores and side effects of treatment were recorded. Both groups received one week of pharmacotherapy, after which group A continued with NSAID-opioid management (WHO stepwise approach) and group B received the NCPB. Although there was a reduction in basal visual analogue scale (VAS) pain scores in both groups, there was no statistical significant difference between the two. This being said, there was significant decrease in opioid consumption in the NCPB group, which was still evident by week four after the procedure with some effect until death (mean survival 51 days).

A similar reduction in opioid consumption was found in another RCT [29], where the average requirement of analgesic medication was lower in the block group, with only one patient not requiring opioids at the time of death. Kawamata *et al.* [28] found a delayed but significant reduction in opioid requirement 4–7 weeks post-block, which subsequently continued to decrease. A recent meta-analysis [4] found that though NCPB did not always eliminate the use of analgesic drugs, it did cause a significant decrease in opioid requirement, with a mean reduction of 40–80 mg/d as well as a reduction in constipation. In contrast, a recent RCT by Johnson *et al.* [31] found no difference in pain scores and opioid consumption in those patients with pancreatic and upper abdominal cancers when comparing pharmacotherapy, CPB, and splanchnicectomy.

### 4.5. Survival and Quality of Life

There has been considerable discussion on the positive effect of pain control on increasing patient survival, yet only a few studies have addressed this issue. Newly diagnosed pancreatic carcinoma patients who present with pain have been linked with a high likelihood of recurrence and impaired survival [32] whether they are amenable to resection or not. An intriguing finding by Staats *et al.* [33] was a significant relationship between improved pain with increased duration of life, suggesting that patients with reduced pain had an increase in longevity. Additionally, the authors found a beneficial effect on mood and quality of life. These findings have been corroborated in other studies; one [19] showed higher mean survival rates in the group with greater pain reduction, and another [34] showed an improvement in quality of life in NCPB groups compared to medical management alone. In contrast, other studies have not been able to reproduce similar results [27,31,35]. In a RCT [28] addressing the influence of NCPB on quality of life, though the results did not show a direct improvement, the authors did find a relative decrease in the deterioration of quality of life attributing this to long lasting analgesic effects and decreased side effects from reduced opioid consumption.

### 4.6. Factors Influencing Outcome

Pancreatic cancer pain can be severe and debilitating, drastically reducing quality of life in patients who already have an attenuated life expectancy. Nearly 75% of pancreatic cancer patients suffer from pain at the time of diagnosis, with more than 90% of patients suffering from pain in advanced disease [4]. Therefore, it is important to recognize factors which influence the efficacy of NCPB and may help improve their quality of life outcomes. A recent retrospective study [38] found that positive outcomes of NCPB were more likely in those patients who had a lower pre-block opioid dose (mean oral morphine equivalent dose of 152.5 mg/day *versus* 357.2 mg/day for negative group, p = 0.02), suggesting less pain correlated with better outcomes. Positive outcomes were also found in those patients who had pain of a shorter duration prior to block and limited sedative administration during the procedure.

#### 4.6.1. Timing

Another factor potentially influencing efficacy is the appropriate timing in the course of disease in which to provide NCPB. Early NCPB has been associated with improved outcomes in the past [18,34]. Ischia *et al.* demonstrated an increased incidence of immediate complete pain relief (p < 0.05) in those patients who underwent NCPB within two months of pain onset. Similarly, Erdek *et al.*'s [38] retrospective study noted positive outcomes in patients who had shorter duration of pain. Another recent study compared early and late sympathetic neurolytic blocks with pharmacotherapeutic intervention in advanced cancer patients. They found that there was an overall improvement in quality of life with a decrease in pain and opioid consumption in the neurolytic group compared with medication management alone, though there were no major differences between the early and late block groups [34].

#### 4.6.2. Technique

Few studies have directly compared the various techniques used to achieve NCPB, and reports are mixed. As mentioned previously, Ischia found no difference in immediate and up to death pain scores between the retrocrucral, transaortic, and bilateral chemical splanchnicectomy groups. In a non-randomized prospective case-controlled study of 59 patients [39] NCPB was compared to videothorascopic splanchnicectomy, which found that both techniques had similar efficacy in pain reduction and decreased the mean daily opioid consumption. NCPB however was found to be associated with significantly improved physical, emotional and social well-being with the added benefit of being less-invasive. In terms of radiologic guidance, Eisenberg [15] found positive short-term outcomes from NCPB regardless of imaging modality used.

#### 4.6.3. Anatomical Location of Disease and Injectate Spread

The efficacy of celiac plexus block is thought to be in part dependent upon the adequate spread of the neurolytic agent used. A retrospective study by de Cicco *et al.* [40] reviewed records of a group of patients who had abnormal anatomy of the celiac area (documented by prior CT) and had undergone CT-guided single-needle NCPB for pain relief. The authors found that there was a close correlation between the block's efficacy and duration of analgesia with the spread of the injectate; with the intuitive conclusion that an inverse relationship exists between the amount of spread and the abnormal anatomical areas. Interestingly, a deficient spread of solution was sometimes noted in unaffected areas and that lack of spread was mainly involving the lower quadrants of the celiac area. Another study [41] comparing efficacy of NCPB in reference to location of pancreatic cancer, *i.e.*, head *versus* body and tail, found that NCPB sufficiently diminished pain in 74% of cancer patient and had greater efficacy in those with cancers located in the head than in the body and tail. However, despite tumor location, the analgesic effects were reduced with advanced spread of disease. Apart from the physical barriers presented by tumor proliferation, other mechanisms are thought to be involved in restricting the potency of NCPB. As mentioned earlier, neurolysis of the celiac plexus will inhibit pain transmission from the viscera. With tumor infiltration of nearby structures, somatic and neuropathic pain mechanisms become increasingly involved, which is thought to explain why in some patients refractory pain increases near the end of life. Post-mortem neurohistochemical examination of the celiac plexus and ganglia subjected to neurolysis in one study reported incomplete destruction of nerve fibers and ganglia after using alcohol for neurolysis [42], which could explain why the analgesic effect of NCPB is often temporary. In an earlier report, some authors of the aforementioned study also proposed placement of a catheter near the celiac plexus for either an intermittent infusion of alcohol or local anesthetic to facilitate long term analgesia, with good results in a small group of patients [43].

### 4.7. Complications

NCBP has been shown to be a relatively safe procedure. Most commonly reported adverse events include local pain (96%), hypotension (10%) and diarrhea (44%) [15]. Eisenberg's [15] meta-analysis reported serious adverse events occurred in 13 of 628 (2%) patients. These included neurologic (lower extremity weakness and paresthesia, epidural anesthesia and lumbar puncture) and nonneurologic (pneumothorax, shoulder, chest and pleuritic pain, and hematuria) complications. Other rare complications reported in the literature include retroperitoneal bleed, urinary retention, gastroparesis, bowel perforation [4,44], anterior spinal artery syndrome, aortic dissection or pseudoaneurysm.

## 5. Additional Therapeutic Modalities

### 5.1. Radiotherapy

External beam and intraoperative radiotherapy have been reported to improve survival [45] and provide pain relief [7] when used alone [46] or in combination with chemotherapy; however, analgesic effects may take several weeks to occur [47]. There have been reports of up to nearly 88% of patients achieving partial to complete pain relief in one case series with radioactive iodine [49], whereas a randomized study [49] has shown improvement of pain in 39% of patients treated with gemcitabine and 6% with 5-fluorouracil. Comparison of efficacy between radiotherapy with pharmacologic or analgesic interventional procedures, or combinations of therapeutic options remains to be assessed.

### 5.2. Intrathecal Therapy

Intrathecal therapy (IT) can be used to mitigate pain in those whom comprehensive medical management (CMM) is ineffective or in those who suffer from its related toxicities. IT has been shown to reduce severe pain in patients suffering from refractory cancer pain, with one study [50] showing a significant reduction of patients suffering from severe pain after receiving neuraxial analgesia (*i.e.*, intrathecal or epidural analgesia). Another multicenter RCT [13] evaluated the efficacy of implantable intrathecal drug delivery systems (IDDS) in comparison to CMM in patients with advanced cancer in which clinical success was defined as achieving ≥20% reduction in pain scores or same score with ≥20% decreases toxicity. The authors reported that 84.5% of the IDDS group and 70.8% of the CMM group achieved clinical success (p = 0.05). The IDDS group had an overall reduction in mean VAS scores by 52% (p = 0.055), a reduction in toxicity scores by 50% (p < 0.05), and improved survival of 54.9% of patients at six months when compared with a 37.2% survival in the CMM group (p = 0.06). This trial also showed that the algorithm used in the CMM group also reduced cancer pain by 39% and reduced toxicity by 17%. The conclusion of the study was that overall IDDS significantly improved analgesia with reduced drug related toxicity.

Various algorithms have been put forth in order to optimize intrathecal therapy [51,52]. In 2007, an interdisciplinary expert panel [52] updated the algorithm for intrathecal agent administration in non-malignant and end-of-life pain. First line agents include morphine, hydromorphone, and ziconotide. Fentanyl was upgraded to a second line agent due its granuloma sparing effect. Local anesthetics (LA) can be added to improve analgesia, with one study showing reduced opioid tolerance development in the LA plus morphine group as opposed to morphine-only group [53]. Addition of gamma-amino butyric acid agonists like baclofen and alpha-2 agonists like clonidine may also be used in conjunction with opioids to control severe neuropathic pain [51]. The panel concluded that there is moderate evidence favoring the use of intrathecal agents for long-term management of moderate to severe cancer pain.

### 5.3. Therapeutic Modalities under Investigation

#### 5.3.1. Vanilloid Receptors

Vanillioid receptors are found in a variety of cells, including cancer cells and activated T cells. When activated, these receptors can alter plasma membrane stability, mitochondrial function, and can induce apoptosis. Vanilloids, such as capsaicin and resiniferatoxin (RTX), also have neurotoxic properties, and act via vanilloid receptor 1 (VR1) on target sensory neurons leading to apoptosis and necrosis. Interestingly, a study [54] found upregulation of VR1 receptors in the pancreas of cancer patients when compared to a normal pancreas, as well as an increase in those cancer patients with higher pain scores than those with lower scores. The authors found that RTX diminished cell growth and induced apoptosis in cancer cells. Thus, vanilloid receptors are novel therapeutic targets in an effort to mitigate cancer and its associated refractory neuropathic pain.

#### 5.3.2. Gene Therapy

Another developing paradigm in the pain management of chronic pancreatic pain is gene therapy. Recently, the effect of direct pancreatic injection of a commonly used experimental viral vector (herpes simplex virus, HSV) carrying the human preproenkephalin (HSV-Enk) gene was tested in animal models of pancreatitis [55,56]. Studies have found both increased met-enkephalin (gene product) in the peripheral nerve terminal endings as well as in the dorsal root ganglion (DRG) at the corresponding levels of innervation of the pancreas (T8-12). These findings were associated with reduced pain behaviors measured in the animals. In addition, the HSV-Enk gene was noted to provide both anti-inflammatory effects and maintain tissue preservation in the pancreas [57]. Though there may be some time before human phase trials are begun in pancreatic cancer patients, targeted gene therapy provides hope for controlling debilitating pancreatic pain in the future.

#### 5.3.3. Phentolamine

Another method to control sympathetically mediated pain (SMP) is by way of phentolamine infusions. Phentolamine, an α-receptor blocker, has been shown to diminish SMP [58-60]. A recent case series [61] studied eight patients, four of whom had pancreatic cancer and the remaining with other abdominal cancers. Of the four pancreatic carcinoma patients, one experienced pain relief till death, one had undergone stenting and thus pain relief due solely to phentolamine could not be determined, and the other two had partial pain relief and underwent repeat phentolamine infusions and required NSAIDS/opioids for improved pain control. Variation in response may be attributed to inadequate dosing in some patients [62] and multiple pain processes occurring as mentioned above. Common complications of phentolamine infusion included hypotension and tachycardia. Well-designed randomized trials are necessary to clearly determine the true efficacy of intravenous phentolamine in pancreatic cancer patients.

## 6. Conclusions

Management of pancreatic cancer related pain can be quite challenging. It might best ideally involve a multidisciplinary approach including pain and palliative medicine specialists, surgeons, and oncologists. The goal of palliative care is to improve quality of life and ease the burden of pain. Optimization of pharmacotherapeutic options to control pain is the initial step towards this goal. Should this fail or the adverse effects of analgesics occur, other therapeutic options are best considered. NCPB has been shown to relieve pain in a selected group of patients. There has been an improvement in study quality throughout the past few decades; however these studies continue to carry several well-described methodological deficiencies including small sample sizes, limited extractable data, and limited comparability by lack of sham procedures or previously validated controls [4]. There remains some dissent among authors with respect to quality of life and with this as a primary outcome assessed in only a few studies; there is a clear need for more validated quality of life measured evaluations [4]. Additionally, comparisons between techniques, efficacy of agents used, optimizing patient selection, and possible synergistic effects with additional therapeutic modalities need to be further explored. At present, the majority of current data supports the use of NCPB. In comparison to standard pharmacotherapy, it has been shown to be more effective in reducing pain and leads to decreased opioid requirements and thus their related side effects. Studies show analgesic effects lasting for one-to-two months, with one review showing up to 92% partial to complete relief after three months [15]. In light of limited life-spans, this duration is considered to be significant. It should be highlighted that NCPB is not meant to be used as a replacement for pharmacotherapy, but rather as an additional therapeutic tool in selected patients. Another effective option to mitigate cancer pain is administering medication via intrathecal pumps. Though available literature supports the use of IDDS, more randomized controlled trials are needed to assess the efficacy and safety of medications, combination of medications, and assess efficacy of various agents by comparative studies. In the near future, experimental treatments such as vanilloid receptor targeted drugs or gene therapy may prove to be effective either as individual or complementary therapeutic options in the management of pancreatic cancer pain. In conclusion, optimal treatment of pancreatic cancer pain requires a global approach to the patient with attention to the many complex origins and mechanisms responsible for pain production, pain potentiation, and quality of life deterioration, followed by a systematic approach to the delivery of a timely, step-wise, clinically validated, and well-integrated multidisciplinary patient care model.

## Figures and Tables

**Table 1.** Common Analgesics for Mild to Moderate Pain [7,11].

**Drug**	**Dose Range (mg)**	**Half life (hours)**	**Max Daily Dose (mg/day)**	**Duration (hours)**
Acetaminophen	500–1000 q 4 h	2–4	4000	
Aspirin	650 q 4–6 h	2–4	6000	
Ibuprofen	200–400 q 6 h	3–4	4200	
Naproxen	250–500 q 12 h	13	1100	
Codeine	15–60 q 4–6 h	2–3	–	3–6
Oxycodone	5–30 q 4 h	2–3	–	3–6
Hydrocodone	Given with non-opioid	2–4	–	3–4

q: every; h: hours

**Table 2.** Commonly used analgesics for Moderate to Severe Pain [7,11].

**Drug**	**Equianalgesic Dose**		**Half-life (hours)**	**Duration of Analgesia (hours)**
	**IM**	**PO**		
Morphine	10	30–60	2–4	3–6
Oxycodone	15	20–30	2–3	3–6
Methadone	10	20	8–59	4–8
Hydromorphone	1.5	7.5	2–3	3–4
Fentanyl transdermal	–	–	–	48–72

**Table 3.** Randomized Controlled Trials: Celiac Plexus Block in Pancreatic Cancer.

**Study**	**Patients**	**Interventions**	**Results**	**Comments**	**AHRQ Quality Score [36]**

Mercadante 1993 [30]	20 pancreatic cancer patients randomized after receiving 1 week of analgesics; 10 received NSAIDS/narcotics, 10 had NCPB via posterior approach	Pts either received analgesic meds and were increased toward goal dosages to obtain VAS <4 ; OR underwent NCPB with 25 ml 75% alcohol bilaterally via posterior percutaneous approach then received analgesics like first group.	Though both groups had significant reduction in VAS, no difference between the two. Significant reduction of opioid use in NCPB group	Randomization method not described, unblinded, small study population Opioid intake reduced for up to 7 weeks post NCPB or until time of death	4.5/10

Lillemoe 1993 [26]	Pts with histologically proven unresectable pancreatic cancer, 72 had placebo, 65 had splanchnicectomy	Intraoperative chemical splanchnicectomy with 20ml 50% alcohol Control: 20 ml NS injection as a placebo	NCPB group: Patients without preop pain had significantly reduced VAS scores and delayed onset of or no subsequent pain Pts with preop pain had both reduced pain and increased survival time.	Randomization method not described, double-blinded Nearly 2/3 of pts with preop pain and relief by NCPB had return of mod – severe pain before death. Data implies 3–4 mo of min to mild pain before return of severe Sx.	8/10
		
Staats 2001 [33]			Increased longevity in NCPB group; significant negative correlation between postop pain and longevity.		8/10

Kawamata 1996 [28]	21 pancreatic cancer pts in palliative care	NCPB: in 10 pts, 8 ml LA + 15–20 ml 80% alcohol Control: in 11 pts, NSAID-morphine. Increase in dose when VAS ≥ 3/10 SQ morphine equivalent given when unable to take orally	VAS scores significantly lower in NCPB group for first 4 weeks, morphine consumption significantly lower in weeks 4–7. Though QOL scores did not differ significantly, they deteriorated only slightly in CBP group	Randomization method not described, unblinded, small study population	5/10

Polati 1998 [29]	Pts with histologically proven unresectable pancreatic cancer, 12 pts underwent NCPB, 12 pts had pharmacotherapy	NCPB: 6–8 ml of LA + 7 ml of absolute alcohol, Control: 6–8 ml of LA + WHO guidelines of pharmacotherapy	Immediate significant pain relief (in first 48 hours) in NCPB group; but long-term results did not differ between two groups. Reduced opioid need in NCPB group at ¼ and ½ survival time (not significant at ¾ survival time) and thus reduced opioid side effects.	Randomization method not described, double-blinded, small study population	6.5/10

Wong 2004 [27]	Pts receiving noncurative pancreatic surgery were eligible with a NRS of ≥ 3/10. 50 pts/group used in analysis	NCPB: 10 ml LA + 10 ml absolute alcohol Control: sham procedure by SQ and IM LA+ pharmacotherapy Rescue blocks if NRS ≥ 6/10 or intolerable opioid adverse effects	Greater reduction in pain scores in NCPB group but no significant difference in opioid consumption, QOL and survival.	Randomization by calling a central telephone number in blocks of 4 pts per group, double-blinded, small study population. Though a greater number of patients survived in NCPB group, results were not significant.	8/10

Zhang 2008 [37]	56 pts with unresectable pancreatic cancer, 29 pts had CT-guided NCPB, 27 treated with pharmacotherapy	NCPB: 5 ml LA + 20 ml absolute alcohol Control: MS contin	At day 1, 7 and 14 VAS lower in NCPB than control; opioid consumption lower in NCPB group. Though both groups improved, QOL not different between two groups	Randomization method not mentioned, unblinded.	6/10

Johnson 2009 [31]	65 pts (57 pancreatic cancer, 3 gallbladder cancer, 1 bile duct cancer, 1 duodenal cancer, 3 unknown); 18 withdrew or died in 2 months	MM: protocol for opioids CPB: “usually alcohol”, done by various operators TS: done by various operators	No difference in pain relief or opioid consumption between the 3 groups.	Multicenter study, Randomization by telephone in blocks of 3 and stratified by treatment center, tumor type, and current opioid status. Unblinded, Small study population. No standardized injectate for CPB described	7/10

Pts: patients, NS: normal saline, LA: local anesthetic, MS: morphine sulfate, WHO: World Health Organization, Sx: symptoms, NRS: numeric rating scale, SQ: subcutaneous, IM: intramuscular, QOL: quality of life, VAS: visual analogue scale, MM: medical management, TS: thoracic splanchnicectomy.
